# The distribution and retention of paclitaxel and doxorubicin in multicellular layer cultures

**DOI:** 10.3892/or.2012.1650

**Published:** 2012-01-20

**Authors:** JOO-HO LEE, KUN NA, SOO-CHANG SONG, JAEHWI LEE, HYO-JEONG KUH

**Affiliations:** 1 Department of Medical Life Science, College of Medicine, The Catholic University of Korea, Seoul 137-701; 2 Department of Biotechnology, The Catholic University of Korea, Gyeonggi-do 420-743; 3 Biomedical Research Institute, Korea Institute of Science and Technology, Seoul 136-791; 4 Divison of Pharmaceutical Sciences, College of Pharmacy, Chung-Ang University, Seoul 156-756, Republic of Korea

**Keywords:** cell cycle distribution, doxorubicin, drug penetration, multicellular layer, paclitaxel

## Abstract

Limited distribution of anticancer drugs has been recognized as a significant hurdle to efficacy. We investigated a detailed penetration/distribution profile of paclitaxel-rhodamine (PTX-rd) and doxorubicin (DOX) in multicellular layer (MCL) cultures of human cancer cells as an *in vitro* model for avascular regions of solid tumors. MCLs were exposed to drugs and fluorescent images of frozen sections were acquired for determination of drug penetration into MCL under various exposure conditions. PTX-rd and DOX showed drastically different profiles of penetration. DOX showed full penetration after 1 h and accumulation over 3 h, whereas PTX-rd showed slow and limited penetration, with accumulation only within the top 20% of layers by 2 h and insignificant penetration even at 72 h. Drug retention in MCL was more dependent on drug concentration, rather than exposure time, i.e., drug distribution increased by 6.3- and 2.5-fold for PTX-rd and DOX, respectively, when exposed to higher concentrations under comparable AUC exposure (1 μM × 24 h vs. 50 μM × 0.5 h). Anti-proliferative activity of PTX and DOX in MCL, as determined by cell cycle analysis, was minimal and may be attributed, at least in part, to their limited distribution in multicellular cultures. Overall, we demonstrated that penetration and retention of PTX and DOX in MCL was not only concentration- and time-dependent, but also schedule-dependent. It is suggested that slow releasing formulations or a slow infusion regimen may not necessarily be desirable, especially for PTX, due to insufficient penetration and accumulation which may result from a low local concentration at the target site.

## Introduction

Most solid tumors show resistance to chemotherapy. To exert efficacy, anticancer drugs should reach their target cells at lethal concentrations. To reach all tumor cells, drugs must penetrate multiple cell layers, which is a significant hurdle imposed by a solid tumor microenvironment. In contrast to normal tissues, solid tumors have a complex microenvironment. The tumor microenvironment consists of various cell types and an enriched extracellular matrix, as well as an abnormal vasculature and hypoxic regions (1–3). Besides poor perfusion, extravascular compartments of solid tumors pose additional conditions that make diffusion of drug through cell layers difficult, due to increased distance between blood vessels and an increased interstitial fluid pressure (1). The majority of studies on drug resistance have focused on the cellular mechanism via genetic alteration (4). Recently, much attention has been paid to the impact of drug penetration and distribution into solid tumor tissues, as shown by a considerable amount of data published on the limited distribution of various anticancer agents and on novel strategies for improvement of drug distribution, which may ultimately result in greater efficacy.

Paclitaxel (PTX) is one of the most important chemotherapeutic drugs used in the treatment of human solid tumors, including ovarian, breast, and head and neck cancers (5). The excessive accumulation of PTX in the periphery of tissue fragments (histocultures) and multicellular tumor spheroids (MCS), with very limited penetration into the interior has been reported (6,7). The limited penetration has been attributed to tissue adhesion (specific and unspecific), cellularity, and expression of P-glycoprotein (P-gp) (6,8,9). Doxorubicin (DOX) is a first line antineoplastic agent against many solid tumors, as well as leukemias and lymphomas (10). Limited availability of DOX due to its insufficient distribution in solid tumors in association with efflux by the P-gp pump, increases sequestration in endosomes and tumor cell packing density (3,9,11,12).

The potential contribution of tissue penetration of PTX and DOX to their limited efficacy has not been fully evaluated. This may be due to the absence of proper methods for *in vitro* investigation of tissue penetration and distribution and cell survival. As *in vitro* models, multicellular layers (MCL) and MCS have become the most commonly used tools for qualitative and quantitative assessment of drug penetration/distribution (3). In MCLs, a drug is added on one side of the MCL and its appearance on the other side is measured by appropriate analytical methods. MCLs have been used successfully in the study of the pharmacokinetics of anticancer drugs, such as tirapazamine and other DNA alkylators (13–16). MCS also demonstrate many of the properties of solid tumors *in vivo*, including expression of extracellular matrix (ECM), tight junctions, and lower cell proliferation to the center, and have been used in examination of the kinetics of drug penetration (17,18).

Earlier studies evaluated penetration of anticancer agents including PTX and DOX in 3D models and the influence of P-gp expression, cell density, and differential expression of the ECM (9,19,20). The effect of the drug exposure schedule, however, has not been fully studied and controversy still exists over the importance of drug concentration vs. exposure duration for increased efficacy (7,21). In the present study, we investigated a detailed penetration/distribution profile of PTX and DOX in MCL of human colorectal cancer cells as an *in vitro* model for avascular regions of human solid tumors. We compared the penetration, distribution, and retention of PTX and DOX in MCL following a long period of exposure up to 72 h as well as under different exposure conditions. Drug penetration and retention in MCL showed not only concentration- and time-dependency, but also schedule-dependency. Our data demonstrate the different penetration kinetics between PTX and DOX, and the relative importance of long exposure time in terms of penetration and retention in multicellular layer cultures. The present study may provide the rationale for the need of pharmacokinetic modulation of drug distribution, which may in turn lead to efficacy modulation.

## Materials and methods

### Cell lines and chemical reagents

The human colorectal cancer cell line, DLD-1, was obtained from the Korea cell line bank (Seoul, Korea). Cells were grown as monolayers at 37˚C in RPMI-1640 (Gibco-BRL, Rockville, MD) supplemented with 100 μg/ml streptomycin (Sigma Chemical Co., St. Louis, MO), 100 units/ml penicillin (Sigma Chemical Co.), and 10% heat-inactivated fetal bovine serum (WelGene, Daegu, Korea) in a humidified atmosphere of 95% air plus 5% CO_2_. Paclitaxel-rhodamine (PTX-rd) was synthesized as described in the previous study (22). DOX was a generous gift from Dong-A Pharmaceutical (Giheung, Yongin, Gyeonggi, Korea). Calcein-AM was purchased from Molecular Probes, Inc. (Eugene, OR) and other reagents, unless otherwise noted, were purchased from Sigma Chemical Co.

### Growth and characterization of MCLs

Exponentially growing cells (3×10^5^) were seeded on a collagen-coated, microporous (0.4 μm) membrane in Transwell inserts (Corning Costar, Acton, MA), as previously reported (22). Cells were allowed to attach for 4 h and the membranes were then submerged in a culture jar supplemented with 150 ml of RPMI-1640 medium with intermittent stirring. MCL were allowed to grow up to 8 days. Frozen sections (20 μm) were prepared in a vertical direction (perpendicular to the membrane) using Tissue-Tek O.C.T compound (Sakura, Torrance, CA):20% sucrose (1:2). Sections were stained with H&E and uniformity of MCL growth was assessed under a light microscope: only MCL batches with uniform growth across the membrane were used for data collection.

### Penetration experiments

MCLs, after 8 days culture were transferred into 24-well plates or 6-well plates, as needed, depending on experiments. At the beginning of the penetration assay, the medium in the donor chamber was replaced with fresh medium containing each agent, i.e., calcein-AM, PTX-rd, DOX, or ethidium homodimer-1 (EthD-1). The medium volume in the top and bottom chamber was 200 and 700 μl in 24-well plates, and 200 μl and 7 ml in 6-well plates, respectively. Under these conditions, fluid levels between the bottom chamber (BC) and top chamber (TC) were even, and, consequently, penetration was driven by the concentration gradient.

For drug uptake in monolayers, 1×10^6^ cells/well were plated in an 8-well chamber slide system (Lab-Tek II, Nalgene, Nunc International, Naperville, IL) and cells were allowed to attach for 24 h. Cells were then exposed to drugs for 3 h and observed under a confocal microscope (Bio-Rad Laboratories, Hercules, CA) at λ_Ex/Em_ of 482/528 nm for PTX-rd and at λ_Ex/Em_ of 480/590 nm for DOX.

### Image acquisition and analysis

Frozen sections of MCLs were examined under a fluorescence microscope (Olympus, AX70, TR-6A02, Tokyo, Japan) at λ_Ex/Em_ of 488/517 nm for calcein-AM and at λ_Ex/Em_ of 482/528 nm for DOX, PTX-rd, and EthD-1. The images of interest were obtained using the DP70-BSW software as an average of 1360×1024 μm size (0.2 μm^2^/pixel) with ISO 200 and exposure time between 1/4–1/30 using intra-group control. Line morphometric analysis of the fluorescence intensity was performed using OPTIMAS version 6.5 (Media Cybernetics^®^, Silver Spring, MD). A minimum signal level just below threshold was set for each tissue section based on an average background reading from regions without staining. Data were normalized for tissue autofluorescence (background) and plotted against the relative distance (%) from the drug exposure side (either top or bottom side) of the MCL. When plotting, fluorescence within 5–10% distance from the membrane was manually deleted in order to eliminate a spill-over effect of high fluorescence intensity from the membrane. Horizontal images were obtained by optical sectioning of MCL using confocal laser scanning setup (LSM 510 Meta, Carl Zeiss, Jena, Germany), which was connected to an inverted microscope (Axiovert 200M, Carl Zeiss).

### Measurement of cell cycle distribution using a fluorescence-activated cell sorter (FACS)

After drug exposure, MCLs were washed with cold-PBS, and treated with trypsin-EDTA (0.05% w/v) on ice for 1 h at 37˚C for 5 min. Cells were then suspended as single cells. For monolayers, cells were collected as a single cell suspension after drug exposure. Cells were then fixed with 70% cold-ethanol and stored at −20˚C until FACS analysis. Upon analysis, fixed cells were washed with cold-PBS, treated with RNase A (50 μg/ml) and PI (50 μg/ml) at 37˚C for 5 min, and then immediately analyzed using a flow cytometer (FACScan, Becton-Dickinson Immunocytometry System, San Jose, CA). Cell cycle analysis was performed using Modifit (Verity Software House, Topsham, ME).

## Results

### Growth of MCL of DLD-1 cells

**Figure 1:**
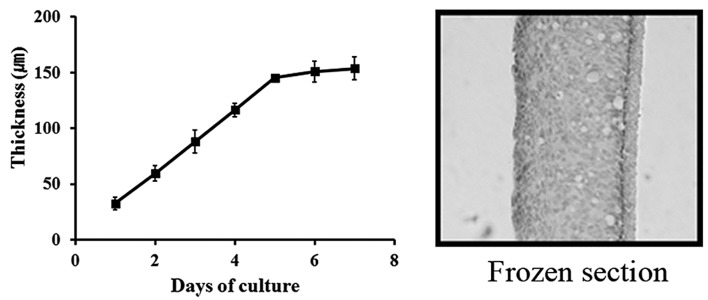
Growth of multicellular layers (MCL) of DLD-1 cells. (A) A growth curve of MCL until 8 days of culture (n=6). (B) Representative frozen sections of MCLs grown for 5 days and stained with hematoxylin (the membrane is shown on the right side).

DLD-1 MCL growth was evaluated for 8 days. The thickness of MCL reached ~150 μm, as observed on frozen sections (Fig. 1). A stable multi-cell layer culture of 15–17 cells was formed with no necrotic part, which appeared to be an appropriate model for avascular regions of human solid tumors.

### Distribution of compounds with different physicochemical properties in DLD-1 MCL

**Figure 2:**
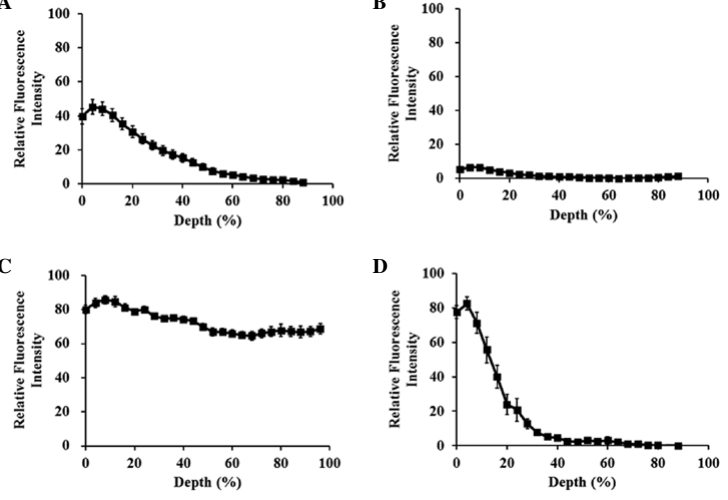
Distribution of four different compounds in MCLs of DLD-1 cells following 2 h of exposure. MCLs were exposed to (A) 40 μM of calcein-AM, (B) 50 μM of PTX-rd, (C) 100 μM of DOX, and (D) 20 μM of ethidium homodimer-1 (EthD-1). Exposure to calcein-AM occured at room temperature, whereas others took place at 37˚C.

P-gp is believed to cause multi-drug resistance (MDR) by reducing drug uptake into cells and tissues. On the other hand, several studies have contradictorily reported a positive effect of P-gp on tissue penetration (3,6,8,11,19). We compared MCL penetration of PTX (50 μM), DOX (100 μM), calcein-AM (a vital dye, 40 μM), and EthD-1 (20 μM) after 2-h exposure in the top chamber of a transwell. All compounds, except EthD-1, are known as substrates of P-gp. Penetration of DOX was complete, whereas PTX showed insignificant penetration, and calcein-AM and EthD-1 showed an intermediate level of penetration with localization within 20 and 40% depth from the exposure side, respectively (Fig. 2). DOX and PTX are both hydrophobic substrates of P-gp, yet, the two compounds showed drastically different profiles of penetration. We selected these two compounds for further investigation of the detailed penetration kinetics.

### Penetration and distribution of PTX-rd in DLD-1 MCL

**Figure 3:**
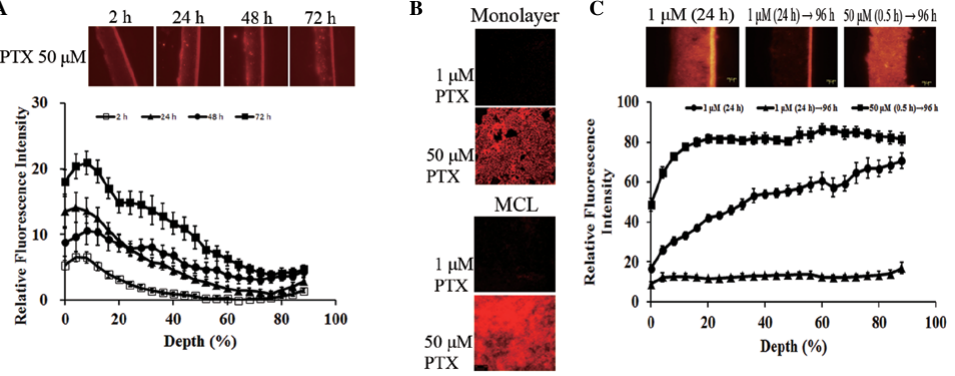
Distribution of PTX-rd in MCL under various exposure conditions. (A) MCL were exposed to 50 μM of PTX-rd up to 72 h. Drug exposure was at the top side of the MCL (0% depth). (B) Monolayers and MCL were exposed to PTX-rd (1 and 50 μM) for 3 and 48 h, respectively. Confocal section of MCL was taken at 60–90 μm (40–60% depth) distance from the top. (C) MCL were exposed to 1 μM for 24 h or 50 μM for 30 min, then further cultured in drug-free media until 96 h. Drug exposure was at the bottom side of the MCL (100% depth). In (A) and (C), fluorescence within the bottom 10% was deleted in order to eliminate over-spill from the membrane.

Exposure of MCL to 50 μM PTX-rd resulted in time-dependent penetration; fluorescence above the background level was observed within 20% in 2 h and within >90% by 72 h (Fig. 3A). Based on the AUC of the fluorescence profile, drug distribution increased by 3- and 6-fold at 24 and 72 h, respectively, compared with that of 2 h. Distribution profiles of 24 and 48 h showed similar AUC; nonetheless, an increased level of distribution in the deeper layer of MCL at 48 h indicated that distribution into deeper layers occurred only after 48 h, and, before that, preferential accumulation of PTX-rd was limited to the upper 40% of the layers, with minimal distribution to the lower 40% of layers.

PTX accumulation was compared between the monolayers and the MCL. Both cultures were exposed to 1 and 50 μM of PTX-rd (Fig. 3B). No fluorescence above the background was observed at 1 μM in either of the cultures. When exposed to 50 μM PTX-rd, the fluorescence intensity in monolayers was maximal at 3 h and the level of intensity was comparable to that of the MCL (optical section at 40–60% depth, 60–90 μm from the exposure side) after 48-h exposure. The significantly longer time taken for MCL penetration, as well as the limited penetration profile, indicated the presence of a penetration barrier for PTX in DLD-1 MCL (Fig. 3A and B).

In order to gain an understanding of the effect of exposure conditions on drug penetration and retention, we compared PTX-rd distribution after exposure to 1 μM (for 24 h) or 50 μM (for 0.5 h), followed by wash-out until 96 h (bottom side exposure, Fig. 3C). PTX showed preferential accumulation at the exposure side (100% depth side) after 24 h exposure at 1 μM. However, the level of fluorescence dropped to less than 20% throughout the MCL depth following incubation in drug-free media until 96 h, which indicated complete wash-out, resulting in no drug retention. Dramatically contrasting data were obtained after exposure to a 50 μM concentration for 0.5 h, followed by a wash-out period, where PTX-rd showed homogeneous distribution throughout the MCL layers with a 6.3-fold higher level of fluorescence intensity, compared with that of 1 μM × 24 h. These data clearly indicate the advantage of higher drug concentration rather than longer exposure time in tissue penetration of PTX. Note that the drug exposure AUC was comparable between these two groups, i.e., 1 μM × 24 h vs. 50 μM × 0.5 h. Note also that drug exposure conditions shown in Fig. 3C differed from those of Fig. 3A due to a 35-fold larger volume of donor compartment, i.e., drug was given in either the top chamber (200 μl) or the bottom chamber (7 ml) for Fig. 3A and 3C, respectively.

### Penetration and distribution of DOX in DLD-1 MCL

**Figure 4:**
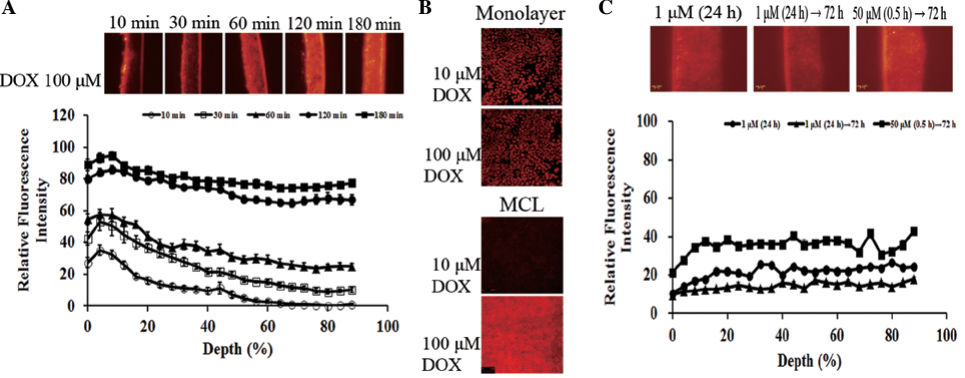
Distribution of DOX in MCL under various exposure conditions. (A) MCL were exposed to 100 μM of DOX up to 3 h. Drug exposure was at the top side of the MCL (0% depth). (B) Monolayers and MCL were exposed to DOX (10 and 100 μM) for 3 and 4 h, respectively. Confocal section of MCL was taken at 90 μm (60% depth) distance from the top. (C) MCL were exposed to 1 μM for 24 h or 50 μM for 30 min, then further incubated in drug-free media until 96 h. Drug exposure was at the bottom side of the MCL (100% depth). In (A) and (C), fluorescence within the bottom 10% was deleted in order to eliminate over-spill from the membrane.

Exposure of MCL to 100 μM DOX resulted in time-dependent drug penetration, as shown with PTX-rd (Fig. 4A). At earlier times (≤30 min), DOX showed preferential accumulation in the upper 40% of the layers, similarly to that observed with PTX-rd. Full penetration was obtained after 1 h and the accumulation level showed a further increase as exposure time increased, up to 3 h. Distribution profiles at 2 and 3 h were similar throughout the MCL depth. Comparison using the AUC of the fluorescence-depth profile showed that drug distribution at 1 and 3 h was 1.5 and 4 times greater than that of 30 min, respectively. Compared with the steep-slope profile of PTX-rd, DOX showed rapid penetration, resulting in a rather flat distribution profile throughout the MCL depth by 1 h (Fig. 3A vs. 4A).

DOX accumulation was compared between the monolayers and the MCL after exposure to 10 and 100 μM DOX, respectively. In MCL, the level of intensity was measured on an optical section of MCL at 60% depth (90 μm from the exposure side). Fluorescence intensity in monolayers was maximal at 3 h for both concentrations. No significant signal was detected in MCL exposed to 10 μM DOX. The fluorescence intensity of the optical section of MCL was comparable with that of monolayers when exposed to 100 μM DOX. The data indicate that DOX accumulation into MCL required a higher concentration, compared with that in monolayers (Fig. 4B).

In order to gain an understanding of the effect of exposure conditions on drug penetration and retention, we compared the distribution of DOX fluorescence after exposure to 1 μM (for 24 h) and 50 μM (for 0.5 h), followed by wash-out until 72 h (the bottom side exposure, Fig. 4C). DOX distribution within MCL was rather flat after 24 h of exposure at 1 μM (compared with PTX, Fig. 3C) and decreased to half-level following 48 h wash-out. Exposure of MCL to 50 μM (0.5 h) resulted in a 2.5-fold increase of DOX distribution in terms of AUC of the fluorescence intensity profile, compared with 1 μM (24 h). These data also indicate the relative importance of drug concentration over exposure time for DOX, as seen in PTX. Note that the drug exposure AUC was comparable between these two groups, i.e., 1 μM × 24 h vs. 50 μM × 0.5 h. Note also that drug exposure conditions shown in Fig. 4C differed from those of Fig. 4A due to a 35-fold larger volume of donor compartment, i.e., drug was given in either the top chamber (200 μl) or the bottom chamber (7 ml) for Fig. 4A and C, respectively.

### Cell cycle distributions following drug exposure

**Figure 5:**
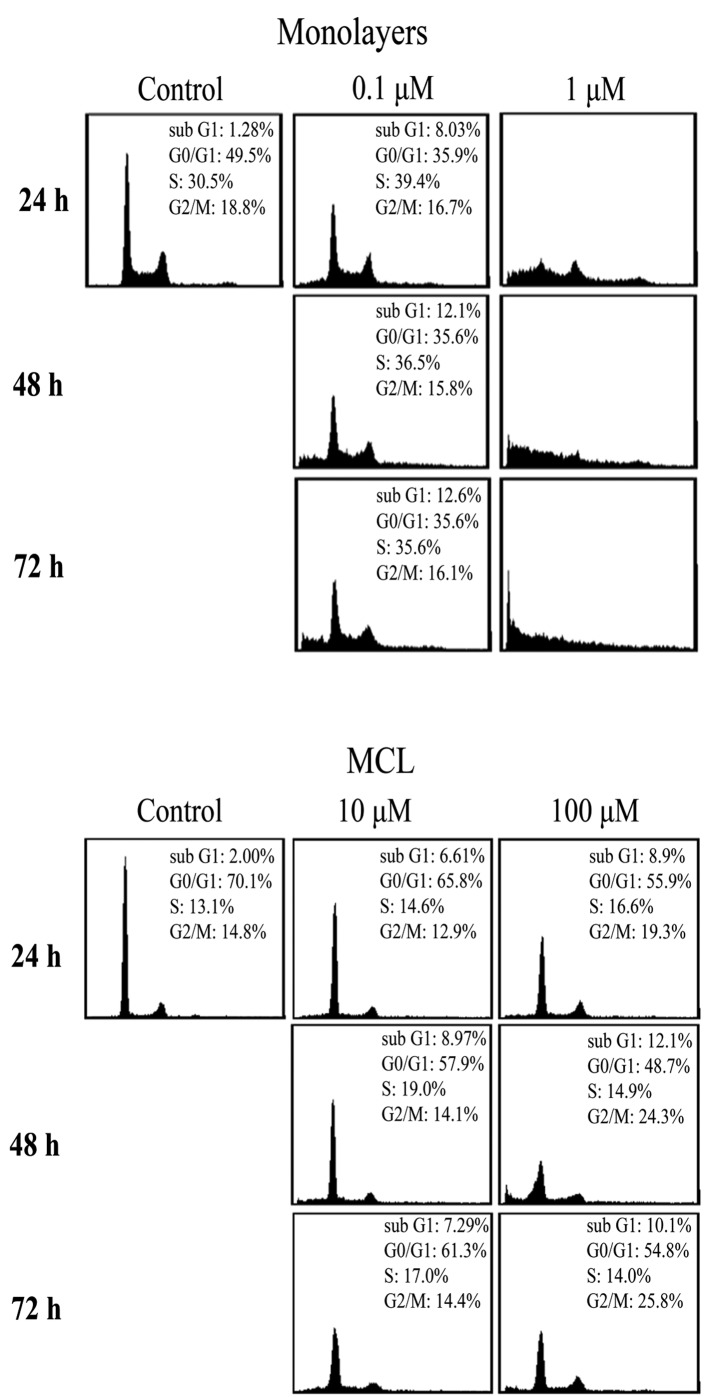
Cell cycle distribution of DLD-1 cells grown as monolayers (A) and MCLs (B) after PTX exposure. Control is non-treated. (A) Monolayers exposed to 0.1 and 1 μM of PTX and (B) MCL exposed to 1 and 50 μM until 72 h.

**Figure 6:**
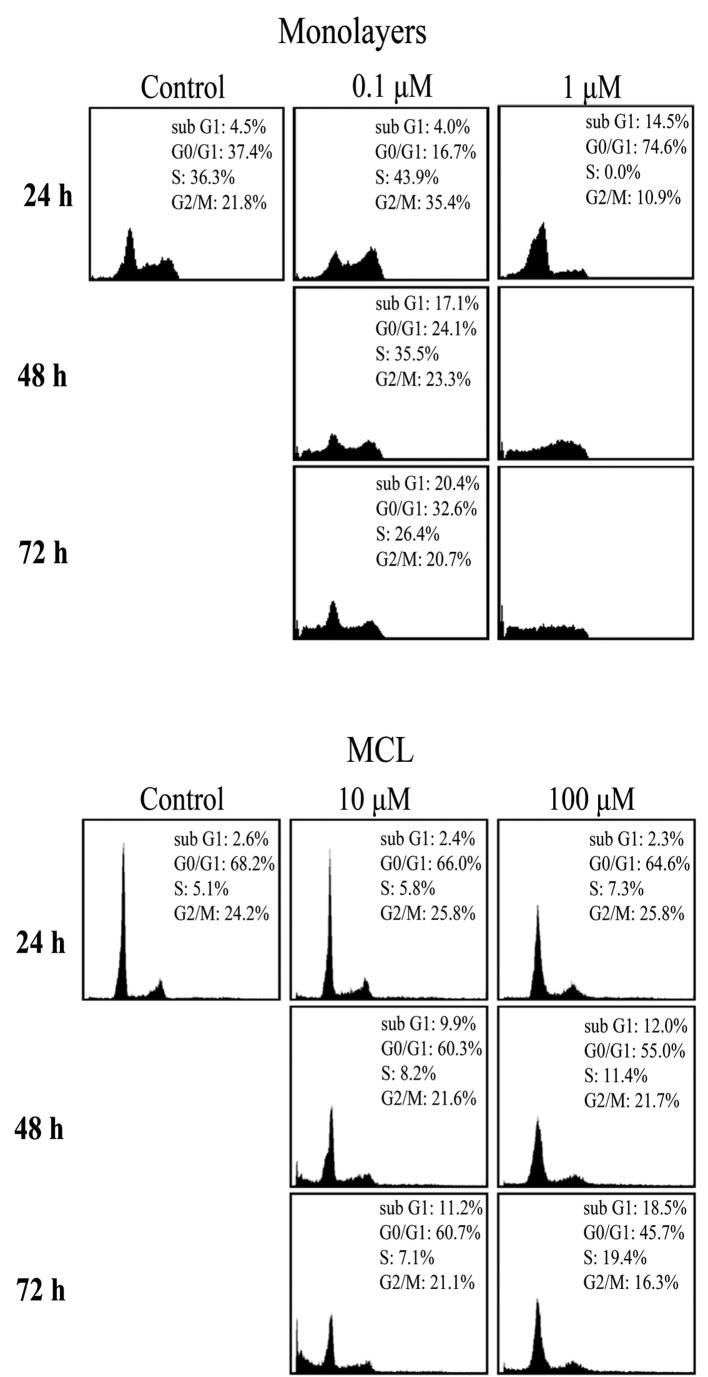
Cell cycle distribution of DLD-1 cells grown as monolayers (A) and MCLs (B) after DOX exposure. Control is non-treated. (A) Monolayers exposed to 0.1 and 1 μM of DOX and (B) MCL exposed to 10 and 100 μM until 72 h.

The limited distribution of PTX and DOX in MCL (Figs. 3 and 4) prompted us to examine its correlation with compromised antitumor efficacy by comparison of the growth inhibition between monolayers and MCLs. DLD-1 cells grown as monolayers showed a typical cell cycle distribution and the distribution was completely destroyed upon exposure to 1 μM (10 × IC_50_) PTX (Fig. 5A). On the other hand, no significant changes in cell cycle distribution were observed in DLD-1 cells grown in MCL, even at concentrations as high as 10 or 100 μM, except for a small increase in the G_2_/M phase after exposure to 100 μM (19.3–25.8%) (Fig. 5B). Note that %G_0_/G_1_ was higher (49.5 vs. 70.1%) in MCL, compared with monolayers, indicating significantly slow cell proliferation. These data combined with the drug distribution profile (Fig. 3) suggest that limited penetration of PTX may contribute to the lack of anti-proliferative activity in DLD-1 cells grown in MCL (Fig. 5). In the same way, changes in cell proliferation were determined following exposure to 0.1–10 μM of DOX in DLD-1 cells grown in monolayers and MCLs. Cell cycle distribution was completely abolished at 1 μM (10 × IC_50_) DOX in monolayers (Fig. 6A). Exposure of DLD-1 MCL to DOX at 10 and 100 μM resulted in a significant increase in the sub-G_1_ fraction (2.3–18.5%) (Fig. 6B); nonetheless, the changes were marginal, compared with that of monolayers (Fig. 6A). Hence, the limited distribution of DOX in MCL also contributed to the limited anti-proliferation activity, as shown for PTX.

## Discussion

Different from agents targeting tumor vasculature, interstitial drug delivery is the main determinant of anticancer efficacy for agents targeting tumor parenchymal tissue. The solid tumor microenvironment plays a critical role as a barrier to interstitial delivery; hence, many studies have focused on defining the major mechanisms for and strategies to overcome interstitial delivery. MCS has been utilized in the study of penetration of anticancer agents, including DOX, vinblastine, PTX, and methotrexate (9,18,23–25). MCL models have been used for a more quantitative study via a direct assessment of drug penetration into multicellular layers (12,14,22,26–28). These multicellular cultures show many characteristics of *in vivo* tumors, including the prevalence of extracellular matrix and presence of hypoxia and desmosomes between cells (9,13,29). If a substance is added to one side of the MCL, the appearance of the substance on the other side is measured for determination of its penetration kinetics (9,12). Penetration kinetics of PTX, methothrexate, 5-fluorouracil (5-FU), DOX, and tirapazamine have been studied using these MCL models (9,14,22,28).

Tumors typically contain irregular, tortuous networks of leaky microvessels with heterogeneous blood flow and intervessel distances that fall between 50 and 200 μm (15–20 cell diameters from the nearest blood vessel), which is significantly greater than that of normal tissue (a few cell diameters) (30,31). In this respect, the DLD-1 MCL cultures used in this study closely represent the avascular region of human solid tumors because 8-day-cultured DLD-1 MCLs showed ~150 μm thickness, with 15–17 cell layers (Fig. 1). We used static chamber conditions, which may also represent the tumor microenvironment of high interstitial pressure and little convection movement. Although quantitative extrapolation may not be possible, a relative comparison should be feasible.

In the present study, we evaluated and compared the penetration profiles of PTX and DOX in DLD-1 MCL. For measurement of time- and dose-dependent distribution within MCL, we used drug fluorescence tracing either by the drug itself (DOX) or by a rhodamine-derivative (PTX). We also compared drug uptake and drug-induced cell cycle changes between cells grown as monolayers and MCL. As previously reported, PTX-rd shows physicochemical and biological properties similar to those of the parent PTX and is considered suitable for both pharmacokinetics and efficacy evaluation (8,22,32,33).

For P-gp substrates, it is well known that cellular uptake decreases in P-gp-overexpressing cells grown in monolayers because substrates are subject to efflux by P-gp after entering cells (19). On the other hand, tissue penetration of P-gp substrates has been reported to increase as shown with BODIPY-taxol in 3-dimensional cultures of P-gp-overexpressing cells (8). When P-gp efflux was inhibited by efflux inhibitors, drug sequestration at the drug exposure side occurred and the availability of the drug to penetrate into deeper layers decreased (19). DLD-1 cells used in the present study are P-gp (+) and penetration of DOX (100 μM) was completed within 1 h. However, the other two P-gp substrates, calcein-AM and PTX-Rd, showed limited penetration, compared with DOX (Fig. 2). These data suggest that penetration of P-gp-substrates is influenced by other factors, such as physiochemical properties of the agents, besides their interaction with P-gp.

We used fluorescence microscopic analysis for drug penetration. Images of representative regions of the MCL sections were acquired and quantitative line morphometric analysis was performed. In this line morphometric analysis, areas adjacent to membranes were intentionally deleted in order to eliminate the spill-over effect, as described in Materials and methods. Fluorescence intensity in the very top layers of MCL showed slightly decreased levels, i.e., the maximum level of fluorescence was observed in 5–10% of layers. This was observed in either case when drug was added to the top or bottom chamber. Reasons for this lower level in the exposure layers may be attributed to drug-efflux by P-gp; however, it may also be due to loss of drug during wash-out for sample harvest. Note that this effect was not observed on the data from the bottom side exposure (Figs. 3C and 4C) due to manual deletion of the spill-over range.

PTX penetration into MCL showed time-dependency (Fig. 3A). Its penetration into whole layers was observed after 48 h and the tissue level increased further until 72 h, suggesting slow penetration. Until 24 h, drug accumulation was limited to the upper layer and insignificant fluorescence intensity was observed in deep layers. The distribution profile was ‘flip-flopped’ at 48 h, i.e., the level in the upper layers decreased, as the level in the lower layers increased, while the AUC of the tissue distribution profile remained the same. Hence, our data suggest that PTX penetration was so slow to show a two-step pattern of distribution, i.e., accumulation during the first 24 h and movement through the layers during the next 24 h. A similar phenomenon has been reported for PTX distribution in histocultures: PTX accumulates in the peripheral part of the tissue (histocultures) and the break-through penetration to the center region is caused by cell density reduction induced by drug-induced apoptosis after 24 h (6,9). As expected from limited penetration of PTX in MCL, cell cycle dysregulation was minimal in MCL, even at the exposure to 10 or 100-fold higher drug concentration compared to monolayers (Fig. 5B).

Contrary to PTX, DOX showed relatively rapid penetration into MCL layers, resulting in homogeneous distribution within 1 h, after which the accumulation level increased and a plateau level was reached at 3 h (Fig. 4A). As DOX showed homogeneous and significant accumulation in MCL within 3 h compared to PTX, growth inhibition measured by cell cycle dysregulation was greater than that of PTX, showing a significant increase in sub-G_1_ fraction (18.5%) by 72 h (Fig. 6B). However, growth inhibition as as measurement of the increased sub-G_1_ fraction was much lower and significantly more delayed than expected. It has been suggested that cells grown in a 3D microenvironment resembling the *in vivo* conditions exhibit a significantly greater resistance to drugs than cells grown as single cell suspension or monolayers (multicellular resistance) (1,34–36). Three-dimensional cultures have fewer of actively proliferating cells, which in turn requires a greater drug concentration and a longer exposure for an effect to occur (37,38). A further study is warranted to measure the actual drug concentration and pharmacodynamics in MCL conditions.

Our data suggest an advantage of high drug concentration over longer exposure time under the same level of exposure (CxT) in tissue accumulation of PTX and DOX (Figs. 3C and 4C). Drug retention in MCL after a long wash-out period (≥72 h) was significantly greater when exposed to a higher drug concentration over a short duration (50 μM × 0.5 h), compared with a lower drug concentration over a long duration (1 μM × 24 h). This effect was much pronounced for PTX compared to DOX, and comparable with the observation made by another group that drug accumulation was much greater when exposed to a higher concentration under the same CxT exposure condition (21,39). This can be attributed to saturation of the P-gp pump by high drug concentration and subsequent intracellular accumulation, leading to apoptosis induction and reduced cell density (9,21,39). Our data may not be compared with a study that suggested a cell kill advantage of longer exposure, in which the cell kill was measured after disaggregation of spheroids into single cell suspension (7).

In this study, we demonstrated that: i) the drug penetration into multicellular layers was not only dependent on the substrate's specificity for the P-gp pump but also dependent on other factors including the physiochemical properties of the drugs; and ii) the penetration and retention of PTX and DOX in MCL was not only concentration- and time-dependent, but also schedule-dependent. It can be suggested that slow releasing formulations or a slow infusion regimen may not necessarily be desirable, especially for PTX, due to insufficient penetration and accumulation which may result from a low local concentration at the target site.
